# Oral vocabulary training program for Spanish third-graders with low socio-economic status: A randomized controlled trial

**DOI:** 10.1371/journal.pone.0188157

**Published:** 2017-11-29

**Authors:** Clara Gomes-Koban, Ian Craig Simpson, Araceli Valle, Sylvia Defior

**Affiliations:** Department of Developmental and Educational Psychology, Universidad de Granada, Granada, Spain; Fordham University, UNITED STATES

## Abstract

Although the importance of vocabulary training in English speaking countries is well recognized and has been extensively studied, the same is not true for Spanish–few evidence based vocabulary studies for Spanish-speaking children have been reported. Here, two rich oral vocabulary training programs (definition and context), based on literature about vocabulary instruction for English-speaking children, were developed and applied in a sample of 100 Spanish elementary school third-graders recruited from areas of predominantly low socio-economic status (SES). Compared to an alternative read-aloud method which served as the control, both explicit methods were more effective in teaching word meanings when assessed immediately after the intervention. Nevertheless, five months later, only the definition group continued to demonstrate significant vocabulary knowledge gains. The definition method was more effective in specifically teaching children word meanings and, more broadly, in helping children organize and express knowledge of words. We recommend the explicit and rich vocabulary instruction as a means to fostering vocabulary knowledge in low SES children.

## Introduction

Vocabulary knowledge is an important aspect in learning to read. In confirmation of this, high correlations between vocabulary and reading comprehension have been repeatedly reported in the literature for English-speaking children [1]. In particular, for at-risk children, such as children with low socio-economic status (SES) or learning difficulties, vocabulary deficits tend to be difficult to overcome and can accompany them throughout their entire academic career [2–4]. This can subsequently bring extra burdens to these students’ already challenging educational path [5,6].

Studies with Spanish-speaking children report a similar pattern of results. According to a recent OECD report ([7]; p. 216), around 12% of variance in reading performance of Spanish children is linked to socio-economic differences. Specifically in regard to vocabulary, it has been reported that differences in vocabulary levels between low and middle/high SES Spanish children are common and that this difference remains throughout the elementary school years [8]. Likewise, a study with Peruvian fourth-graders has shown that reading literacy was significantly correlated to children’s SES and vocabulary knowledge levels [9].

Based on the strong body of evidence regarding the effects of vocabulary intervention for English-speaking children in the elementary school years, research summary reports (e.g., [10–12]) and books for educational practitioners (e.g., [13,14]) recommend that vocabulary should be taught by providing rich and varied language experiences, by explicitly teaching individual words and word-learning strategies, and by fostering word awareness. Additionally, the teaching methods used should promote active processing of the words and enable multiple encounters in order to boost learning outcomes.

Interestingly, these recommended teaching methods are still not common practice at the elementary school level in Spanish-speaking countries. Based on our observations in some schools, one procedure consists of giving teachers lists of words (e.g., [15]) that children should learn in each primary grade. As a consequence, vocabulary knowledge, although recognized by the teachers as an important skill, is sometimes treated as a component of reading comprehension that does not need specific instruction, and the teaching of vocabulary is confined to writing definitions of words (mostly pre-selected from the text book) after reading a text passage.

While there is a disconnect between research and practice in many countries (e.g., [16]), in the case of Spain, one of the reasons for this gap is the fact that educational policies are still not strongly guided by evidence. Some researchers in Spain, along with the Ministry of Education, have attempted to compile important findings related to reading research in the form of local and national reports [17–19]. However, most of these reports are largely based on results of studies with English-speaking populations. This can be problematic, as English and Spanish differ in many aspects, including in areas related to the transparency of the orthography [20], prosodic features [21,22], as well as differences in speech production [23] and the rate of learning to decode from print [20]. In fact, in the case of transparency, Share [24] has argued that English is an outlier orthography and that common models of reading developed using evidence gathered from English-speaking participants are “ill equipped to serve the interests of a universal science of reading” (p. 584). Consequently, it is plausible that teaching vocabulary in English and Spanish may require different strategies. Additionally, the language differences identified have been associated with tuitional practices (e.g., [25]). Thus, it is possible that practices in the classroom between Spain and English-speaking countries need to be adapted due to differences in the attitude and practices of parents related to supporting their children’s literacy development at home. For these reasons, there is still a need to generate more evidence supporting theoretically-motivated models which are applicable to Spanish-speaking populations, taking into account environmental and language-specific differences.

In the area of vocabulary, the lack of theoretically motivated, comprehensive evidence-based training programs to foster vocabulary knowledge in Spanish-speaking children is quite apparent. To the best of our knowledge, there are only three published studies that have examined vocabulary training in Spanish-speaking children [26–28]. In the first study [26], Spanish pre-school aged children were taught just five words and all were from the same semantic category. In the second study with Spanish fifth-graders [27], although a larger set of training words were used, no random allocation of children to groups was undertaken. Instead, the training and control groups were composed of children from different schools. Thus, it is possible that uncontrolled differences between the two schools could partially explain the results reported. Finally, the third study with Chilean kindergarten children [28] does mention the use of a randomized assignment and a control group. Nevertheless, procedures and statistical analysis are poorly reported and, consequently, the results are hard to interpret.

In sum, although the basic underlying developmental and cognitive mechanisms of acquiring vocabulary may not differ between Spanish and English-speaking children, linguistic, cultural, school-related, and parental practice issues may influence the effectiveness of teaching methods. Thus, it cannot simply be assumed that a method which has been found to be effective in English will also have the equivalent effect in Spanish, and the efficacy of any method for improving vocabulary on Spanish-speaking children must be empirically determined in well-designed studies. Accordingly, the present work was designed to investigate the effects of two methods of oral vocabulary training in a sample of Spanish children. Such research would also have the potential to trigger discussions among educators in schools about more effective ways of fostering vocabulary development of elementary-school-aged children.

In English-speaking countries, in which the importance of fostering vocabulary using evidence-based teaching methods is more recognized, there are still researchers who argue that there are too many words to be taught explicitly (which is the recommended technique). This is mostly due to the assumed complexity of word knowledge [29] and the observed number of words learned along with the pace of word learning in the school years [30,31]. However, this does not necessarily mean that explicit teaching cannot operate as an additional learning channel alongside others, such as incidental learning [32], learning word meanings from context [33], wide reading [34], and reading aloud [35], all of which have also shown their value in supporting vocabulary development.

Moreover, in the case of at-risk children, comparative studies point to an advantage of explicit teaching of vocabulary for kindergarten children [36] as well as for elementary school children [37]. These authors argued that the encounters with words and text experienced by children with reading difficulties, low motivation to read, and poor language environment will not be equally productive or necessarily lead to vocabulary gain in comparison to average achievers. In accordance with this view, Perfetti [38] claims that children with comprehension difficulties will be able to learn fewer words during their reading experiences than children with well-developed reading comprehension skills. Therefore, in order to try to raise the level of at-risk children’s vocabulary knowledge to that of average achievers, explicit and systematic vocabulary training at a young age is recommended [39].

The first step in order to develop effective and systematic vocabulary training is to try to understand and describe word knowledge and what it means to know a word. As a starting point, one could define vocabulary as the number of words a child possesses in his/her mental lexicon, no matter how superficial the knowledge is. This is known as breadth of vocabulary. However, a large amount of research undertaken in the area of vocabulary acquisition suggests that such a simple definition is not adequate (e.g., [29,40–43]). A description of the qualitative aspects of word knowledge, such as its richness (e.g., polysemy, derivation), structure (semantic fields and links), and relation to other knowledge should also be considered. Collectively, these factors can be denoted as the depth of vocabulary knowledge. Importantly, it has been argued that vocabulary depth and the efficiency with which knowledge about words can be accessed have more influence on the higher-order processes in reading comprehension than does vocabulary breadth [44]. In accordance with this idea, the lexical quality hypothesis [38,43] suggests that high-quality mental representations of word knowledge are responsible for facilitating the access and integration of word knowledge when reading text. The quality of these mental representations of word knowledge would be determined by linguistic knowledge (phonological, orthographic, morphological, syntactic, and semantic) and knowledge stability (consistent and reliable retrieval), its synchrony of activation (integration of all knowledge components), and its fast access. In summary, although theories regarding vocabulary acquisition may vary in some aspects, they tend to agree that vocabulary knowledge is complex and develops incrementally, and reading comprehension is supported by both broad (quantity) and deep (richness) word knowledge.

In practical terms, this means that vocabulary intervention programs should be comprehensive and aim to not just increase the number of words that children recognize, but also to grow depth of word knowledge, that is, its quality, recall promptness, and correct use. One example of such a comprehensive evidence-based vocabulary intervention program for explicit teaching of vocabulary to elementary school children is the “rich vocabulary instruction” developed by Beck and colleagues [13]. This type of instruction is based on concepts of repeated exposure to words, deep processing of word meanings, and retrieval practices. Additionally, these authors stressed the importance of using “student-friendly” word definitions, that is, definitions specially modified to be focused and easily understood by the students, as well as being embedded in anchor sentences. This rich instruction has repeatedly shown positive effects on the knowledge of taught words [45,46], as well as transfer learning effects on control words and reading comprehension of passages containing the words [47–49]. Interestingly, a rich extended version of instruction which additionally included activities aimed at motivating children to use the taught words beyond the classroom, and so, indirectly fostering *word awareness*, was particularly useful in bringing about significant improvements in story comprehension. Word awareness is a term used in the field of vocabulary research to refer to *metalinguistic knowledge* of words [50], and is defined as awareness of, interest in, and appreciation of words [51]. It has been argued that the correlation between vocabulary and reading comprehension can be partially explained by metalinguistic awareness [52]. However, evidence about the effects of word awareness on vocabulary knowledge and reading comprehension in controlled settings is still scarce and more studies are needed to understand the mechanisms regarding how this metalinguistic knowledge specific to words affects word learning and reading comprehension [53].

Apart from comprehensive vocabulary training programs, various studies investigating the effects of single teaching methods for promoting vocabulary development have been of central importance to guide the development of effective and systematic vocabulary training for elementary school children.

Stahl and Fairbanks’ meta-analysis [54] reported that teaching methods using definitional or contextual emphasis produced large effects and were similarly effective in teaching the meanings of target words and in using the words correctly in *cloze-*type sentences (sentences in which the target word is left blank for the student to complete; [55]). For comprehension of text passages containing the trained words, a small advantage for a combination of both definitional and contextual methods was found. In contrast, learning transfer effects captured by standardized measures of vocabulary and reading comprehension not containing the taught words were, in general, small.

Similar results were reported in the more recent meta-analysis carried out by Elleman and colleagues [53]. Of note, these authors found that vocabulary instruction had a larger impact on customized rather than on standardized measures of vocabulary and reading comprehension. Their conclusion was that the existing standardized measures of reading comprehension may not be sensitive enough to capture changes related to vocabulary training.

An important systematic review of vocabulary instruction was given in the influential National Reading Panel report [10]. Based on analysis of data trends, some of the recommendations for vocabulary teaching practices were: (a) teach vocabulary indirectly and directly; (b) provide repetition and multiple exposures to words; (c) provide rich contexts for words; (d) actively engage children in tasks; (e) combine teaching methods. Similarly, the relatively new research synthesis developed by the National Reading Technical Assistance Center [12] advocates for frequent exposure to words, explicit instruction, and engaging and interactive activities.

Nash and Snowling [56] directly compared definitional and contextual methods in a sample of 7 to 8-year-olds with poor vocabulary knowledge. The definition program consisted of reading aloud pre-determined, simplified dictionary definitions and asking children to think of a personal experience in which the word would fit. The context program consisted of presenting the word of the day in a short text passage. Children were asked to find words that “would give clues to the meaning of the new word” and write them down in a semantic map. At the end of the context program, children were also asked to think of a personal experience in which the words would fit. Both groups significantly improved their knowledge of the taught words. Nevertheless, only the context group maintained the gained word knowledge three months later. Nash and Snowling [56] concluded that the semantic representations built through the context method were more persistent, well-specified, and stable. Due to its potential to teach children how to independently find out the meaning of words beyond the teaching program, the contextual method was recommended over the definitional. The positive effects of contextually based vocabulary instruction for third-graders was also found by Nelson and Stage [57]. This training involving teaching multiple meanings of words using information embedded in a context brought about significant gains in reading comprehension, especially for children with low initial vocabulary knowledge.

In contrast, Jenkins and colleagues [58] found that a definitional method was more effective to teach children word meanings than a contextual method. In this study, the definition method was “richer,” as in addition to the direct word definitions, it provided two examples for each target word in sentences. In this case, the provision of a “student-friendly context” to the words could have boosted the effects of the definition methodology. The conflicting results between Nash and Snowling [56] and Jenkins and colleagues [58] suggest that, despite these studies both looking at “definitional” and “contextual” methods, there were probably some important underlying differences in how these methods were implemented. Indeed, Beck and McKeown [59] point out the problem with attaching specific labels to training methods, given that under the same name very different teaching concepts can be found. Thus, a detailed examination of individual aspects under teaching methods is imperative in order to enable a more precise interpretation of the methodology’s effect.

One common aspect that is present in the effective vocabulary interventions reported above is that they all include a high level of discussion about and around words. This is consistent with the idea that, as oral vocabulary knowledge develops before children start learning to read, oral language comprehension could form a base for the later development of reading comprehension [60]. This would mean that at the point of learning to read, a child’s ability to derive word meanings from context would extend from oral to written language [34]. In an intervention study with children with reading comprehension difficulties, Clarke and colleagues [61] contrasted an oral language training, a text comprehension training, and a combined oral-text training. In the vocabulary component of the oral language training, graphic organizers (semantic maps), verbal reasoning tasks, mnemonics and illustrations to support the multiple-context learning approach were used. Results showed that the long-term gains achieved in reading comprehension were significantly higher for the children in the oral language training only group compared to both the text comprehension only group and the combined oral language and text comprehension training group. These authors concluded that improvement in children’s oral vocabulary was the main mediator of the positive effects of the oral language training on reading comprehension.

According to Graves [31], listening to and speaking about words can be a great source for vocabulary learning at all ages. Nevertheless, reading instruction usually focuses on practicing to decode from print in the beginning elementary years (mostly high frequent and already known words), and only slowly moves towards strongly emphasizing comprehension of words and content from written text in later years [62]. Thus, a rich oral vocabulary training for children in the transition years, which would correspond to third- or fourth-grade in Spain, could potentially provide these children with extra support in boosting their word learning, and thus assisting children to better cope with the emerging higher reading comprehension demands.

Considering this background, the present study was designed to investigate the effectiveness of two oral vocabulary training programs especially developed for Spanish-speaking children. We had two main goals: (1) to determine if either of two different types of rich learning experiences would be effective at increasing vocabulary knowledge beyond what might be gained through classroom reading activities that did not include explicit vocabulary instruction and (2) to explore if learning transfer effects to items not taught and standardized tests not containing the taught words could be produced by indirectly fostering word awareness in the children. We hypothesized that these two programs based on rich instruction would be equally effective and promote significantly more vocabulary knowledge gains of the taught items in comparison to the control group. In relation to transfer and more broad effects of the trainings, for non-taught equivalent items, an advantage was expected for the context methodology, as this combined word awareness, encountering words in stories, and eliciting word relations. Further, if the effects of both definition and context methods on word awareness were sufficiently strong, both training groups would show statistically significant gains in the standardized measures of receptive and expressive vocabulary at both post-tests beyond that of the control group. Finally, in relation to the reading comprehension measure, again a possible advantage for the children in the context group was expected, as activities allowed extra experience in encountering and manipulating text passages and stories.

## Method

### Participants

Three public schools located in low SES neighborhoods were selected based on recommendations from teaching professionals working in the region. Rather than selecting children based on some pre-determined set of criteria, we wanted the study to have high external validity that potentially better captures the reality in the classrooms as well as to adhere to inclusive educational practices [63]. For these reasons, no screening procedure was performed apart from the grade constraint.

The study was approved by the project’s ethics committee. Signed participation forms were received from the principals of all three schools who participated in this study. Data was only collected from children who had returned signed parental consent forms. The final sample consisted of 100 third-graders (58 boys, 42 girls) from five third-grade classes with a mean age of eight years and two months (range 7;5–9;6) at the commencement of the study. Ninety-six children were native Spanish-speakers and the remaining four children were Spanish language learners (Arabic native speakers). Three children were receiving extra tutoring classes in specific subjects as part of the program “alumnado con necesidades específicas de apoyo educativo” [students with specific educational support need] and two children were attending the program “alumnado con necesidades educativas especiales” [students with special education needs].

### Design

Children within classes were randomly assigned to one of the two training groups, or to the control group. Following this random allocation, due to the higher number of boys in some classes, some girls were randomly selected to be reassigned to ensure that both genders were represented in all groups. Also, for ethical reasons, we ensured that the four Spanish language learners were randomly allocated to one of the two experimental groups only (two children in the context and two in the definition group). Thus the final allocations were definition group (*n* = 33; 13 girls), context group (*n* = 34; 15 girls) and control group (*n* = 33; 14 girls). Assuming that the training methods were effective, we expected to find medium to large effects for knowledge of the taught words (see meta-analysis [53]). A priori power calculations using G*Power [64] indicated that the overall size of the sample and the well balanced nature of the design ensured sufficient statistical power was present for the planned group main comparisons and the expected effect size in the vocabulary knowledge of taught words (with power set to 0.8, *f* = 0.32, equivalent to *η*^2^ of 0.09 and to Cohen’s *d* of 0.64).

Children’s evaluation assessments took place at the beginning of the school year just before the intervention started (pre-test), immediately after the intervention ended (post-test 1), and five months later at the end of the school year (post-test 2). Except for the reading comprehension test, which allows group testing, children were tested individually within the schools in multiple sessions no longer than 30 minutes each.

Evaluations were performed by nine research assistants. The recruited assistants were final year undergraduate students undertaking a “Teacher Education” university program and who had experience in teaching children at elementary school age. Training sessions for the evaluations were held for all research assistants together and it included written material with information about planning, schedules, research ethics, and the instruments’ application rules and answer sheets.

### Measures

The following measures were taken at all three time points:

#### Receptive vocabulary

The Spanish version of the standardized Peabody Picture Vocabulary Test (PPVT-III; [65]) was used. In this test, the child selects one of four pictures to match a spoken word in meaning. Unlike in English, the Spanish version of the PPVT-III does not have two parallel forms, so children were tested at all time points with the same items.

#### Expressive vocabulary

The standardized Vocabulary subtest from the Spanish version of the Wechsler Intelligence Scale for Children IV (WISC-IV; [66]) was used. In this task the child was required to define orally a list of words.

#### Reading comprehension

The standardized multiple-choice test Comprensión Lectora de Complejidad Lingüística Progresiva [67] was used. The third-grade version with two parallel forms takes around 30 minutes and consists of 21 items organized in four main tasks that address comprehension at sentence and short text levels. The texts consist of a group of sentences connected by a common topic and characterized by simple grammatical structures and topics common to children’s experiences at this age. The tasks involve interpreting the meaning of a sentence by marking another sentence that has an equivalent meaning, demonstrating the literal understanding of a short text passage by identifying the main characters and their actions, or showing inferential understanding of concepts not explicitly mentioned in the text by marking statements about the text as ‘true’ or ‘false’. Form A and Form B were used to control for test-retest effects.

#### Knowledge of taught and control words

Consistent with the theoretical concept of incremental word knowledge [29,40,42], a self-report measure of vocabulary knowledge (VK) was used to estimate children’s knowledge of taught and control words. Our VK task contained 30 words (15 words selected at random from the 60 taught words in the training methods, plus the 15 untaught control words). The final list of 30 words was the same for all children. Analogous to the vocabulary subtest from WISC-IV, the VK task consisted of asking children to explain orally the meaning of the words. Children’s answers for each of the words were written down by the examiner and were later scored by two independent raters using a scale from zero to four points according to their correctness and quality (Table 1). The correctness of the definitions was judged based on the entries of the target words in three age-appropriate pre-selected dictionaries. Inter-rater reliability at pre-test (κ = 0.79, *p* < .001), post-test 1 (κ = 0.73, *p* < .001), and post-test 2 (κ = 0.76, *p* < .001) pointed to an acceptable scoring classification system [68,69]. Also the criterion-related validity analyses of the instrument showed acceptable results, with moderate correlations at pre-test between the VK and the WISC-IV Vocabulary subtest, *r* = .58, *p* < .001, and between the VK and the PPVT-III, *r* = .58, *p* < .001 [70].

**Table 1:** Evaluation scale to score children’s answers in the measure of knowledge of taught and control words.

Score	Description of equivalent word knowledge	Example of answer
0	Has never heard the word; No knowledge about the word.	“I have never heard this word before.”/ “I do not know.”/ “It does not sound familiar to me.”
1	Has heard the word, but does not know what it means; Has false concept of word; Children expressed themselves in a manner in which the meaning/intention was not clear.	“I think I have heard it before.” / “Yes, I have heard this word, but I do not know what it means.”/ “I do not know what it is.”/ “I cannot explain it.”/ “I do not know how to explain it.”
2	Has heard the word; General concept of positive/negative; Superficial or incomplete knowledge; Cannot give a general definition using synonym or other words to explain the word; Gives an example repeating the word without additional information, which would explicitly signal meaning knowledge; Definition AND example, but only one is correct (contradictory).	“It has to do with…”/ “I am not sure, but maybe …”/ *amenaza* [threat]: “Someone threatens someone.”
3	Has heard the word; Knowledge restricted to a context/ strongly context bound; Either a correct general definition OR a correct example which includes explicit information that demonstrates meaning knowledge.	*interminable* [never ending]: “A story that never ends.” /*superar* [to surpass]: “If someone gets points in a game and someone else gets higher points.”
4	Has heard the word; Broader and richer knowledge; Definition AND example and both are correct (without contradiction).	*vértigo* [vertigo, dizziness] “Afraid of heights, for example, climb a mountain and be afraid of falling or something.” /*pendiente* [^1^pending, ^2^earring, ^3^slope, ^4^to look after, ^5^to pay attention]: “pay attention to something, and earring, for example, this child is paying attention in class or she wears beautiful earrings.”

## Procedure

### Selection of words

The words to be taught in the intervention, along with control words, were extracted from children’s books adequate for the age group and selection was based on a number of criteria: type (“tier two” words, as defined by Beck and colleagues [13]), grammatical class (adjective, verb, and noun [8]), frequency (medium frequency [71]), productivity (number of derivate forms), and richness (number of definitions). Both productivity and richness were calculated based on the entries for the target words provided in three pre-selected dictionaries appropriate for elementary school children. From the final list of 75 words, 60 were randomly selected to be taught in the intervention (S1 Appendix) and the remaining 15 served as control words (S2 Appendix). Analysis showed that taught and control words did not differ significantly in regard to length (*t*[73] = -1.17, *p* = .247), frequency (*t*[73] = -0.45, *p* = .650), richness (*t*[73] = 0.46, *p* = .649) or productivity (*t*[73] = 0.83, *p* = .409).

### Training sessions

Based on the principle of distributed practice [72], the intervention consisted of twenty sessions with three sessions per week over a seven week period plus a final closing session. In each session, except for the closing session, three words—one verb, one adjective and one noun—were to be taught in small groups of four to nine children (as children were randomly assigned to groups clustered in classes, group size varied according to class size). However, due to unforeseen changes to school schedules during the intervention phase, three training sessions had to be cancelled. The nine words that were planned to be taught on those days were transferred to the sessions that followed. This meant that in the first eight sessions the teaching plan of three words per session was followed. In the remaining nine sessions, four words per session were taught. For both the control and training groups, each session lasted 50 minutes. The small group sessions were held in separate rooms within the school. Thus, it was not possible for the children to observe or hear directly what was happening in a different intervention group.

For ethical reasons and because the implementation of a waiting list null control group was not viable in this project, children in the **control group** were offered an alternative intervention. Thus, the sessions for the control group consisted of reading aloud to the children combined with craft work related to the story being read. The reading aloud was based on the TWA approach (Think before, think While, think After reading), which has shown some benefit for English-speaking children [73,74] and is considered a traditional method found in Spanish books commonly used by elementary schools teachers (e.g., [75]). The story books used were the same books from which the taught and control words were originally selected. Thus, children in the control group were exposed to the same words as the children in the training groups, but the control group children did not receive any explicit teaching of word meanings. This is consistent with the research question of whether these methods would provide an advantage over the standard approach to reading instruction in Spanish classrooms.

Each session for the two **training groups** was divided into three parts: warm-up (part I), core program (part II), and recall game (part III). The activities in Parts I and III were identical for both groups. In Part I, the words of the day were introduced in a motivating and playful manner with a short activity lasting about ten minutes. The main goal was to get children involved, motivated, and curious about the words. Additionally, this first encounter was to give children the opportunity to acquire orthographic and phonological information from the visual and auditory inputs presented. Part III was based on the idea of retrieval practices [72]. It also lasted around ten minutes and consisted of recall games aimed at strengthening the recall and retrieval pathways for the newly learned words.

For Part II, a longer time slot of 30 minutes was reserved. According to McKeown and colleagues [49], choosing the most appropriate vocabulary teaching method depends on the specific instructional goals. As stated in the introduction, in this project we had two main objectives. The first was to enhance depth of vocabulary knowledge by providing rich learning experiences. To achieve this, the core intervention was based on several teaching principles, including repeated exposure to materials in various contexts [13], deep processing [76,77], modeling [78], and scaffolding [79,80]. The second goal was to explore learning transfer effects by indirectly fostering word awareness. It was hoped that the core activities would furthermore have the potential to motivate children to be curious about words, to enjoy playing with and investigating words, their usage, multidimensionality, nuances of meaning, and interrelatedness [13,31]. Additionally, to encourage the children to think about words outside of the intervention sessions, a few extra homework activities were included. Examples of these activities included, for example, asking children to find out what the longest word is in Spanish, to ask their parents what their favorite word was, to write down the first word they heard when they woke up on the following day.

As the focus of the intervention was on oral vocabulary, all activities required an oral response from the children. Specifically, children were asked to pay attention to information presented in written form on a paper or poster, or in a picture (visual), or to listen (auditory), then think, and lastly explain or tell something to the group (oral).

#### The definition method

**Fig 1:**
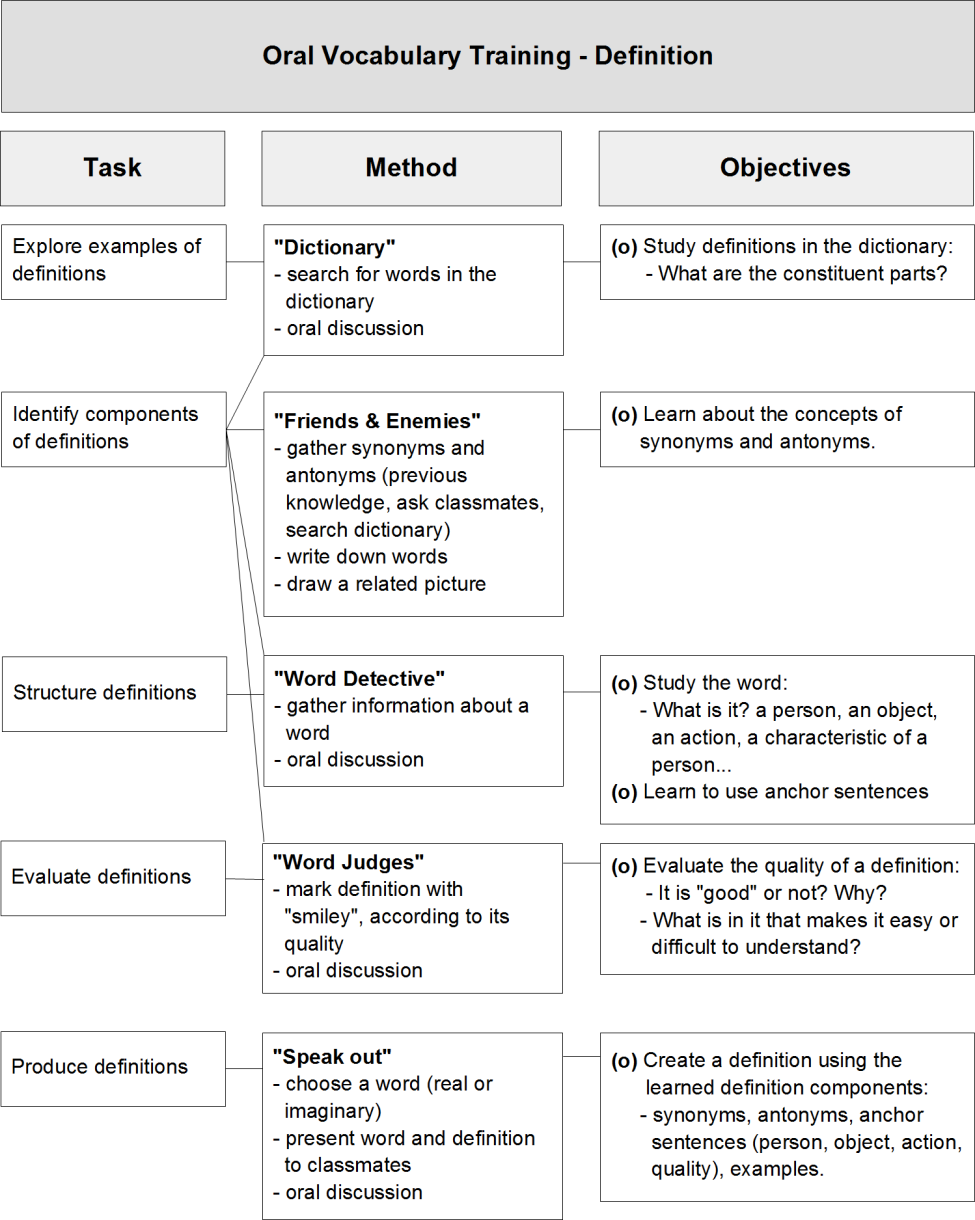
Summary of tasks, methods and objectives in the definition training.

This method involved the direct instruction of dictionary like definitions of words. The central idea was that words were presented and treated in isolation. The focus of the activities was the definitions of words themselves in the sense that children were taught what the components or characteristics of a “good” definition are. High-quality definitions were defined as the ones that are effective in helping others to understand the meaning of an unknown word. The main role of the trainer in this group was to call children’s attention to the structure and components in word definitions, such as synonyms, antonyms, anchor sentences, and examples. Fig 1 shows a summary of the activities used in the definition training and their main goals.

#### The context method

**Fig 2:**
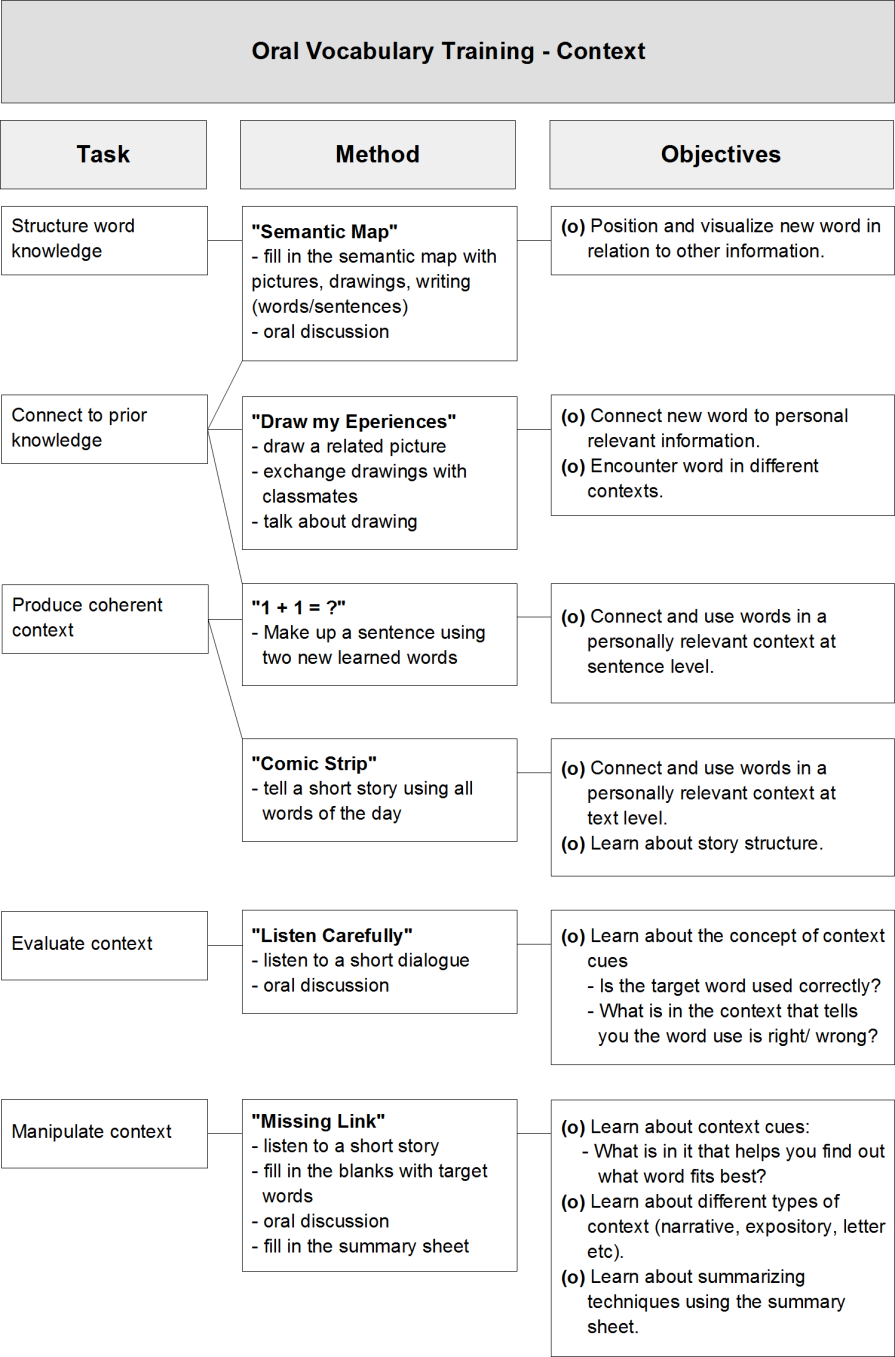
Summary of tasks, methods and objectives in the context training.

In this method the taught words were always embedded in a short text or dialogue. The most important aspect was that the trainer should try not to give a direct and explicit dictionary-like definition of the words being taught at the onset of each session. Rather, children were encouraged to formulate their own verbal definitions based on the information encountered in the presented contexts, in the discussions with the trainer and their group mates, and also by taking advantage of their prior experiences and background knowledge. The main role of the trainer was to help and guide children in building and structuring their own word knowledge network, using their own words and personally relevant experiences. Thus, the activities were designed to explicitly foster the connection between incoming information and prior knowledge. Fig 2 shows a summary of the activities used in the context training and their main goals.

For both training methods, in each session just one of the activities listed was used to teach the words of the day. The sequence of activities followed the order pictured in Figs 1 and 2, and this cycle was repeated until the end of the intervention.

The same nine research assistants who performed the pre-, post-, and follow-up evaluations (as evaluators) delivered also the seven-week training program (as trainers). To avoid contamination, each research assistant was randomly assigned to a single training method. Thus, although some taught more than one group of children, each assistant taught only one intervention condition (either control, definition, or context). Additionally, due to the fact that each research assistant served both as trainer and evaluator, we ensured that the research assistants did not evaluate the children they taught in the intervention sessions, in order to avoid bias at post-test.

Training sessions were held for the research assistants of each intervention method and control group separately to avoid cross-contamination. The training material included a short summary of the main ideas of the project, research ethics, general instructions for the intervention program, a plan of the activities including a description of each task (objective, duration, materials needed, procedure), as well as instructions on how to fill in session protocols. As part of the training, recruited instructors were additionally advised about strategies for improving behaviour in the classroom [81,82].

### Implementation fidelity

Session protocols were completed by the trainers after each session as an additional control to ensure training method compliance (content fidelity). It included a description of what words were taught, what activities were performed during a particular session, how much time each of the activities took, and additional comments about the behavior of the children and other incidents or interruptions. As an additional measure of integrity, structured observation protocols were filled out by a trained third-party observer (quality of delivery). Due to resources and time constraints, only a randomly selected number of sessions were observed. Both these structured protocols as well as the protocols completed by the trainers were reviewed by the principal investigator each week to ensure adherence to the protocols and to promptly identify any potential problems.

In order to offer ongoing support to the trainers during the implementation of the intervention, meetings with all assistants were performed on a regular basis. Just as in the initial training sessions, separate meetings were held for the assistants from each teaching method to avoid contamination. In these meetings, assistants could exchange experiences and discuss any problems encountered in the sessions in relation to using the materials and in dealing with children’s behaviors.

### Statistical analyses

The recommended method to assess the relative effectiveness of training studies is mixed design ANCOVA that takes into account pre-test variation between children [83]. Thus, for each set of results reported an ANCOVA analysis was performed with one between-subjects factor, Group (definition, context, control), and one within-subjects factor, Time (post-test 1, post-test 2), with pre-test scores entered as covariates. The ANCOVA paradigm was implemented as a linear mixed effect model using the lme4 package [84] in the R environment [85]. Consequently, the reported coefficients (*b*s) represent the estimate of the difference between two groups being compared. Exact *p* values cannot be calculated for these types of analyses and the significance of parameters must be assessed by inspecting the confidence intervals [86]. Confidence intervals which contain zero indicate a non-significant parameter–that is, the difference between the two values being compared is not significant. Finally, vocabulary knowledge of the taught and control words was analyzed at the individual item level, rather than at the subject level to simultaneously account for the crossed random effects of participants and items [87]. This technique also minimizes the impact of missing data. Accordingly, for the analyses involving the taught and control words the *b* values represent the difference in the mean VK rating scores, *per item*. For other analyses the *b* values represent the difference in subject means. In sum, in the main analyses that follow, what is being compared is whether there is a significant different between groups on each task, after controlling for pre-test differences.

## Results

### Descriptive analyses

Preliminary analysis, including missing values analysis, showed that assumptions required for the statistical models used were met to an acceptable degree. Table 2 shows the mean score and range for all measures, broken down by time point and group. Omnibus ANOVAs confirmed that there were no significant differences between groups on any of the tasks at pre-test. Of note, the proportion of children falling below the 50th percentile at pre-test was 64% for receptive vocabulary, 82% for expressive vocabulary and 72% for reading comprehension. This pattern of results is in line with previous results reporting a link between SES levels and literacy performance in Spain [7,8].

**Table 2:** Mean (SD) scores and ranges for all measures, broken down by time point and group, along with the results of omnibus ANOVA analyses comparing the group means at pre-test.

Measure	Time Point	Definition Group (*n* = 33)		Context Group (*n* = 34)		Control Group (*n* = 33)		ANOVA
		Mean (*SD*)	Range	Mean (*SD*)	Range	Mean (*SD*)	Range	*p* value
**Taught Words**	Pre-test	20.7 (8.37)	2–34	23.2 (6.63)	8–34	20.2 (7.34)	8–37	.250
	Post-test 1	28.2 (5.87)	10–41	29.1 (8.33)	5–40	23.4 (7.91)	8–35	
	Post-test 2	34.1 (8.14)	16–50	33.9 (8.25)	14–49	29.7 (9.92)	10–48	
**Control Words**	Pre-test	16.4 (7.19)	1–32	16.8 (6.56)	3–30	15.1 (7.17)	2–28	.636
	Post-test 1	19.9 (5.82)	9–34	20.5 (7.01)	5–30	17.1 (6.79)	4–25	
	Post-test 2	25.8 (8.39)	9–45	23.2 (7.72)	10–42	21.4 (8.52)	7–40	
**Receptive Vocabulary**	Pre-test	91.9 (20.78)	56–141	87.9 (20.39)	42–126	90.7 (13.56)	62–118	.693
	Post-test 1	96.7 (20.89)	61–145	96.6 (19.56)	48–142	94.2 (17.79)	54–130	
	Post-test 2	105.8 (21.73)	62–154	100.5 (18.74)	58–135	100.9 (17.64)	69–134	
**Expressive Vocabulary**	Pre-test	12.9 (6.45)	2–27	12.7 (6.76)	2–28	13.0 (5.47)	6–26	.982
	Post-test 1	14.6 (6.80)	2–26	13.4 (5.97)	1–27	13.3 (4.64)	5–24	
	Post-test 2	17.3 (7.27)	5–38	15.4 (6.86)	2–30	16.6 (6.58)	3–32	
**Reading Comprehension**	Pre-test	12.3 (4.70)	5–21	13.2 (4.77)	6–21	12.9 (2.95)	7–18	.982
	Post-test 1	14.1 (3.37)	3–20	13.9 (3.50)	4–20	13.7 (3.64)	5–19	
	Post-test 2	15.0 (3.09)	7–20	14.7 (2.74)	10–21	14.5 (3.90)	3–19	

*Note*. Values reported for the standardized tests of receptive (PPVT-III) and expressive (Vocabulary WISC-IV) vocabulary and reading comprehension (CLP) are raw scores.

### Implementation checks

Based on the implementation data collected, content fidelity was considered sufficient. Nevertheless, trainers reported difficulty in managing children’s behavior. Thus, additional behavior management training was provided and extrinsic motivational strategies were introduced from session seven onwards in order to try to minimize the negative effects on learning outcomes. For example, a list of behavior ground rules was developed in collaboration with the trainers. Accordingly, children could collect stickers won after each session if they were compliant to the behavioral rules.

### Training effects

A summary of the regression coefficients for all analyses can be found in Table 3. Regarding the effectiveness of the training methods, for the taught words at post-test1 both the definition group (*b* = 0.31, *SE* = 0.12, 95%CI [0.08, 0.53]) and the context group (*b* = 0.40, *SE* = 0.12, 95%CI [0.17, 0.62]) demonstrated significantly more knowledge about the taught words compared to the control group. Furthermore, there was no significant difference between the two training methods (*b* = 0.09, *SE* = 0.11, 95%CI [-0.14, 0.31]), suggesting that both training methods proved equally effective at improving vocabulary knowledge of the taught words.

**Table 3:** Regression co-Efficients (*b*) and confidence intervals (95% CI) for models predicting knowledge of taught and control words, as well as for models predicting performance in standardized literacy measures.

Measure	Time Point	Defintion vs Control		Context vs Control		Context vs Definition	
		*B*	95% CI	*b*	95% CI	*b*	95% CI
**Taught Words**	Post-test 1	0.31*	[0.08,0.53]	-0.40*	[0.17,0.62]	0.09 *	[-0.14,0.31]
	Post-test 2	0.25*	[0.03,0.48]	-0.17	[-0.05,0.40]	-0.08 *	[-0.30,0.15]
**Control Words**	Post-test 1	0.19	[-0.01,0.39]	-0.28*	[0.08,0.48]	0.10 *	[-0.10, 0.30]
	Post-test 2	0.25*	[0.05,0.45]	-0.07	[-0.13,0.27]	-0.18 *	[-0.38,0.02]
**Receptive Vocabulary**	Post-test 1	-1.02	[-6.98,4.95]	-4.71	[-1.26,10.60]	5.73 *	[-0.21,11.67]
	Post-test 2	1.28	[-4.74,7.29]	-1.72	[-4.25,7.70]	0.44 *	[-5.55,6.44]
**Expressive Vocabulary**	Post-test 1	1.11	[-1.28,3.50]	-0.77	[-1.62,3.16]	-0.34 *	[-2.71,2.03]
	Post-test 2	0.15	[-2.25,2.56]	-0.85	[-3.24,1.54]	-1.01 *	[-3.39,1.38]
**Reading Comprehension**	Post-test 1	0.13	[-1.24,1.51]	-0.62	[-1.99,0.75]	-0.76 *	[-2.14,0.63]
	Post-test 2	0.12	[-1.28,1.51]	-0.25	[-1.65,1.16]	-0.37 *	[-1.79,1.06]

Note

* indicates significant differences based on the confidence intervals. To derive the parameters and confidence intervals for the context vs definition column, all models were rerun changing the reference category from the control group to the definition group.

At post-test 2 the definition group but not the context group demonstrated significantly more vocabulary knowledge than the control group (*b*_definiton_ = 0.25, *SE* = 0.12, 95%CI [0.03, 0.48]; *b*_context_ = 0.17, *SE* = 0.11, 95%CI [-0.05, 0.40]). Despite this, the difference between the two training methods did not reach significance (*b* = -0.08, *SE* = 0.12, 95%CI [-0.30, 0.15]).

It should be noted that both the covariate of pre-test knowledge, as well as the interaction of pre-test knowledge with Time were significant in these analyses, indicating that children with higher pre-test scores improved more at both post-test 1 and post-test 2 compared to children with lower pre-test scores (and this is also true of all subsequent analyses). This result extends evidence of Matthew effects in reading beyond the oft-cited reports among English learners [88] and underlines the necessity of controlling for pre-test knowledge.

Turning now to potential transfer learning effects involving the control words, at post-test 1 the context group (*b* = 0.28, *SE* = 0.10, 95%CI [0.08, 0.48]) demonstrated significantly more word knowledge than the control group. In contrast, the confidence interval indicates that the difference between definition group and the control group just failed to reach significance (*b* = 0.19, SE = 0.10, 95%CI [-0.01, 0.39]). As for the taught words at post-test 1, for the control words there was no significant difference between the two training methods (*b* = 0.10, *SE* = 0.10, 95%CI [-0.10, 0.30]). At post-test 2 the definition group but not the context group demonstrated significantly more vocabulary knowledge than the control group (*b*_definiton_ = 0.25, *SE* = 0.10, 95%CI [0.05, 0.45]; *b*_context_ = 0.07, *SE* = 0.10, 95%CI [-0.13, 0.27]). Nevertheless, the advantage shown by the definition group over the context group just failed to reach significance (*b* = -0.18, *SE* = 0.10, 95%CI [-0.38, 0.02]).

We next carried out similar analyses for the two standardized measures of vocabulary. With respect to receptive vocabulary, there was no effect of the training methods, either at post-test 1 (*b*_definiton_ = -1.02, *SE* = 3.04, 95%CI [-6.98, 4.95]; *b*_context_ = 4.71, *SE* = 3.05, 95%CI [-1.26, 10.96]) or post-test 2 (*b*_definiton_ = 1.28, *SE* = 3.07, 95%CI [-4.74, 7.29]; *b*_context_ = 1.72, *SE* = 3.05, 95%CI [-4.25, 7.70]). In fact, the only significant factor in determining later receptive vocabulary knowledge was the covariate pre-test vocabulary knowledge. There were no differences between the two methods either at post-test 1 or post-test 2. Regarding expressive vocabulary, there was no effect of the training methods, either at post-test 1 (*b*_definiton_ = 1.11, *SE* = 1.22, 95%CI [-1.28, 3.50]; *b*_context_ = 0.77, *SE* = 1.22, 95%CI [-1.62, 3.16]) or post-test 2 (*b*_definiton_ = 0.15, *SE* = 1.23, 95%CI [-2.25, 2.56]; *b*_context_ = -0.85, *SE* = 1.22, 95%CI [-3.24, 1.54]). Again, the only significant factor in determining later expressive vocabulary knowledge was the covariate pre-test vocabulary knowledge, and there were no differences between the two training methods either at post-test 1 or post-test 2.

Finally, with respect to reading comprehension, no effect of either training method was found, either at post-test 1 (*b*_definiton_ = 0.13, *SE* = 3.04, 95%CI [-1.24, 1.51]; *b*_context_ = -0.62, *SE* = 0.70, 95%CI [-1.99, 0.75]) or post-test 2 (*b*_definiton_ = 0.12, *SE* = 0.71, 95%CI [-1.28, 1.51]; *b*_context_ = -0.25, *SE* = 0.72, 95%CI [-1.65, 1.16]). As for the standardized measures of vocabulary, the only significant factor in determining later reading comprehension was the covariate pre-test reading comprehension. There were no differences between the two training methods either at post-test 1 or post-test 2.

## Discussion

This study set out to test the efficacy of two rich oral vocabulary training methods in comparison to a read-aloud control group in a sample of third-grade Spanish speaking children from schools located in low SES neighborhoods. As expected, below average scores in the standardized measures of vocabulary and reading comprehension were found for children in this sample, and this is in accordance with the literature with English- and Spanish-speaking children [5,6,8].

The main results of the intervention, which corroborate the large body of evidence about vocabulary instruction for English-speaking elementary school children, confirm the high effectiveness of rich instruction [10,12,45–49,53,54,56,58]. It is important to note that the children in the control group were incidentally exposed to the training words in the books that they read and this may have allowed some incidental learning of the training words to occur within this group [26,67,89]. Indeed, reading aloud has been shown in the past to be effective in improving vocabulary learning [90] due to children’s ability to implicit learn words from context. In this sense, we effectively stacked the deck against ourselves, potentially making it more difficult to find statistically significant differences between the training groups and the control group. Nevertheless, using such a control group does have an advantage compared to using a null control group. By using a read-aloud activity for the control group, we were able to determine whether our training methods provided meaningful gains compared to the children’s usual reading activities. Considering these results, we conclude that both definition and context methods of rich vocabulary instruction were more effective in teaching children the meaning of the target words when assessed at the end of the intervention in comparison to the simple exposure to the words that the control group received during their story reading sessions–that is, compared to activities children would likely undertake in reading classes.

Furthermore, five months after the intervention had terminated, children from the definition method still demonstrated a significant learning advantage over the control group. In contrast, the word knowledge advantage shown by the context group over the control group was no longer significant. This suggests that the positive effects of the contextual method boosted word learning only while it was being applied. At the same time, the results suggest that the definition method provided persistent improvement in word knowledge. This pattern of results is contrary to our expectations and those reported by Nash and Snowling [56].

One possible explanation for the long-term advantage of the definition group lies in the methodology itself and its adequacy for this age group. Developmentally speaking, the children who participated in the study were at an age where children in general are just starting to develop their metalinguistic abilities (around 8 years old; [91]). This is supported by informal observations in some activities at the beginning of the study–for example, even when children could correctly judge whether a definition was “good,” or whether a word was used correctly in context, they nevertheless often struggled to express the reason why they thought so. Thus, reflecting about one’s language choices and expressing word knowledge in the form of a general decontextualized definition appeared to be very challenging for these children at the onset of this study. Because the activities in the definition method were designed to clearly identify the relevant elements of a definition as well as to teach how to anchor and structure definitions, they provided children with additional support in organizing and expressing the word knowledge being acquired. In other words, in addition to accumulating new semantic knowledge, the children in this group were learning how to better express semantic knowledge in the form of a clearly structured definition by following an explicit model. In contrast, in the context group, although children were exposed to more words and stories compared to children in the definition group, the manner in which this knowledge was added to the already existing knowledge structures was less prescribed and less systematic. Consequently, these children had to rely more heavily on their own learning strategies for organizing the knowledge being presented.

Moreover, the way in which the activities were designed in the context condition meant that the success of this method was more dependent on the ability of the trainer in moderating the discussions and personal stories told by the children. As a result, even though children in the context group were able to express some of the attained word knowledge in the short-term, this knowledge may have been poorly anchored and was potentially attached to unstable structures that did not facilitate retention and accumulation of further word knowledge in the long-run. This suggests that for children with poor vocabulary knowledge or learning difficulties, a methodology that additionally guides word learning by providing a clearer model of word definition might be more suitable. A similar argument was made by Sternberg [92], in which an elaborated and rich pre-existing knowledge was said to facilitate further learning. It would, therefore, be plausible that a teaching method less dependent on children’s own word learning strategies to accommodate knowledge to already existing (in this case potentially poor) knowledge structures would be more beneficial to children with low vocabulary knowledge. Consequently, the clear (pre-determined) structure and explicit models of student-friendly definitions offered and trained in the definition method used in this work would be more adequate to support these children’s word learning processes.

In relation to the potential of the training methods to produce learning transfer effects to items not taught in the sessions, compared to the control group, only the children from the context group showed significant higher levels of knowledge for the control words immediately after the intervention. That said, the advantage for the definition group over the control group just failed to reach significance [95%CI -0.01, 0.39]. Given this confidence interval, a more practical interpretation of the definition result is that it too was more effective in improving word knowledge than the control method of mere exposure.

The success of the context method in demonstrating transfer effects is in accordance with the predictions: in addition to indirectly fostering word awareness, it was designed to elicit word relatedness and to allow children to encounter a larger number of words within dialogues and stories. This combination of effects would potentially increase the probability of acquiring knowledge about words not taught in the intervention. Nevertheless, we predicted an advantage for the context method over both the control and the definition group at post-test 1. However, no such advantage was found. Interestingly, five months later, a similar pattern to that seen with the taught words was found with the control words. Children in the definition group showed significantly higher levels of knowledge of words not taught in the intervention compared to the children in the control group, but the advantage shown by the context group had all but disappeared. According to the theory of spreading activation [93], improving representations of related concepts, as in the case of words not directly taught in an intervention, could be facilitated by a well-organized word knowledge. As stated previously, the definition methodology seems to have been more adequate in helping children to better structure and express their word knowledge—and crucially, both the gains in semantic knowledge and the ability to better express this knowledge appear to be persistent, long-term gains.

The long-term advantage of the definition method for the non-taught items could be interpreted in two additional ways. The first possibility refers to the effect of word awareness as a means of boosting word learning beyond the intervention sessions. If this method was effective in making children more curious about and attentive to words in general, it is plausible that children could improve their vocabulary knowledge of the control words as well as the taught words. If that were the case, statistically significant gains in the standardized measures of receptive and expressive vocabulary in children pertaining to the definition group would be expected. However, no such differences were found between the groups for either of the standardized vocabulary measures, suggesting that none of the methods had a significant impact on word awareness.

The second explanation for the long-term advantage of the definition method for the non-taught items involves the already mentioned general effect of the definition method in enabling children to express their word knowledge more precisely. If this were the case, statistically significant improvements would be expected in favor of the definition group in the WISC-IV vocabulary subtest, which similarly demands the ability of defining words orally. Yet, no differences were found in this measure. This raises the question about why improvements were found in the VK test of non-taught words, but none were found in the standardized test of expressive vocabulary, which also contained non-taught words. One possibility is that the VK test is more sensitive than the WISC-IV, both in terms of the items used and the scoring scale. In the first instance, all words in the VK test were age appropriate as they were taken from age-appropriate books. In contrast, the WISC-IV is designed for use with a wide age range (6 to 16 years old). Consequently, the first words in the WISC-IV (e.g., vaca [cow]) are probably too easy for the majority of children in this sample while the last ones (e.g. locuaz [loquacious]) are almost certainly too difficult. Consequently, these items would have very low power to discriminate within the sample, and in effect, a reduced number of items would be responsible for most of the variation found in the WISC scores. Accordingly, this would reduce the sensitivity of the test. The second factor to consider is the difference in scale between the two measures. The theory-based VK test employed a five-point scale and therefore would allow for the capture of smaller changes in word knowledge. In comparison, the WISC-IV vocabulary subtest uses a three-point scale (not known/more or less known/known).

Finally, for reading comprehension, no significant differences were found between any of the groups. This is in accordance with the literature, which has shown that vocabulary instruction has a larger impact on customized rather than on standardized measures of vocabulary and reading comprehension [10,36,53,54]. Although, some studies have reported increases in reading comprehension after vocabulary training [48,49], the texts used in these studies were conceived for the intervention and contained the taught words. In contrast, in this study none of the trained words appeared in the reading comprehension standardized tasks. In this sense, more general effects from vocabulary training to reading comprehension were targeted. The results suggest that the training methods were not robust enough in fostering children’s word awareness to make a significant contribution to increasing performance in the reading comprehension measure. It should be noted that, while the hypotheses regarding the direct effects of the intervention were based on clear empirical evidence regarding the effect size [53], the hypothesis regarding the impact on reading comprehension (and indeed expressive and receptive vocabulary) were more theoretically based, and without clear empirical evidence for a specific effect size.

If we consider the theories about the relationship between vocabulary knowledge and reading comprehension, there are specific possibilities in which reading comprehension could be improved via a vocabulary training program. Firstly, if a direct relation is assumed, in the sense that knowing more words in a text would facilitate its comprehension, then improved comprehension could be achieved by teaching the specific words that come up in the tested texts (as it was the case in [48,49]; and in studies of text readability, e.g., [94]). Conversely, if the possibility of an indirect relationship between vocabulary and reading comprehension is assumed, the intervention would need a design that triggers not only an enrichment of vocabulary knowledge, but also a reorganization or restructuring of linguistic and metalinguistic information within the lexicon of the child. This could be accomplished in basically two ways. Either the intervention is designed to teach an enormous amount of words that would, in effect, accelerate or provide additional support of the natural process of learning words (as per developmental theories; [95]) or the specific teaching method must be thought to have the potential to trigger these restructuration processes even when fewer words are taught (as was the hope in this study). The study by Clarke and colleagues [61] did report significant improvements in standardized tests of reading comprehension by teaching the same number of words as were taught in this study. Nevertheless, apart from differences in sample characteristics (children with reading difficulties) and methodology (training length, intensity), in Clarke’s study [61] the oral vocabulary training was only one component of a broader intervention for oral language abilities. One could argue that this intervention had the potential to promote only part of the effects triggered by the more comprehensive oral language intervention employed by Clarke and colleagues. Thus, the results of this study suggest that perhaps a small effect might exist, but this study did not have the power to detect it.

In summary, the results of this study demonstrate the effectiveness of explicit vocabulary instruction based on oral language activities using a sample of Spanish-speaking children from low SES. This is consistent with the works of Beck and colleagues [45,48,49], Nash and Snowling [56], Jenkins and colleagues [58], and Clarke and colleagues [61]. More specifically, the present results suggest that the rich oral vocabulary instruction based on the definition method was more effective to teach target word meanings. Moreover, children appeared to additionally profit from long-lasting and specific effects of the training in regard to structuring and expressing their word knowledge more precisely.

### Limitations and future directions

An important issue to be considered when interpreting the overall results of the intervention is related to children’s behavior. There were children in the sample who displayed disruptive behavior and some trainers reported that the strategies provided in their training sessions were not enough to create an optimal learning atmosphere. In response to this, additional behavior management strategies, based on extrinsic motivation, were introduced from the seventh session onwards in order to try to minimize the negative effects on the learning process. A description of cases would go beyond the scope of this work, but it is relevant to say that considering the numerous class disruptions experienced during the first half of the intervention, the positive effects found in this study are impressive, and are a reason to believe that children are highly skilled word learners when they are exposed to a rich language environment. Furthermore, even though behavioral issues were theoretically covered in the training provided to the evaluators, based on reviewing the protocols and from the weekly meetings, we decided to provide additional training and behavior management techniques to the trainers. When working with populations with behavioral problems, recruiting specialized and more experienced teachers is recommended. An alternative would be to implement more extensive training, along with a trial phase to allow trainers to get to know the children and gather initial experience using the behavioral strategies in the group before intervention officially starts.

A second limitation is that, due to the schools cancelling some sessions, the original plan to teach three words per session had to be modified to teach four words per session. This meant that less time could be spent teaching each of the words of the day, and children had less time and fewer opportunities to talk about each word. Especially in the case of the context method, it has been reported that this teaching technique would require more practice time to produce similar word learning effects compared to the definition method [58]. Potentially, this could have reduced the effectiveness of the two training methods for the second half of the study.

Despite the mentioned limitations, this study nevertheless fills an important gap in the literature, as to the best of our knowledge, it is the first evidence-based vocabulary training program undertaken with Spanish-speaking children which has used a randomized controlled design. Additionally, the inclusion of the five-month follow-up evaluation allowed us to assess the long-term efficacy of the two methods, and the importance of this was highlighted by the fact that the results changed from post-test 1 to post-test 2. Such data not only allow for more accurate cost-benefit estimations of potential interventions, but also enable a deeper understanding of the specific learning effects potentially triggered by the particular teaching techniques. The inclusion of the session protocols as a means of accessing implementation fidelity also allowed us to identify potential problems and take corrective action.

Further qualitative analyses of children’s answers are planned. These should serve as basis for generating hypotheses for future exploration of the positive effects of the definition method in relation to fostering concept formation as well as a self-teaching strategy when learning new words or expressing word knowledge. The clear structure taught using this method could have the potential to help children when further learning new words independently, as they could possibly be trained to take special note of specific information and to develop word storage mechanisms that are supported by a pre-structure. The approach may be especially valuable for children with language comprehension difficulties and with below average vocabulary knowledge, as these children may benefit from scaffolding of more explicit teaching methods. From the perspective of teaching practices, this is also a more straightforward method and easier to apply for less experienced teachers, who might rely more strongly on instructions given in a manual.

Finally, various actions were planned and undertaken to facilitate the dissemination of these results to a wider, non-academic audience. These included oral presentations in the participating schools, written summaries for parents, and a vocabulary program manual in Spanish with detailed description of all activities in the training groups for elementary school teachers (a short summary in Spanish can be found in the supplementary materials; S3 Appendix). However, it is important to say that we are aware of the limitations of research and sample representativeness as well as the complexity and dynamics of school reality. Therefore, by no means it is a claim that the definition vocabulary program, as it was used in this work, is the best program for Spanish-speaking children at this age. In order to inform policy, stronger evidence based on larger scale studies and systematic mapping of vocabulary teaching practices in Spanish-speaking countries is needed. What can be said, based on the experiences gained implementing this project, is that the explicit rich vocabulary instruction can be recommended over one of the traditional methods practiced in Spanish schools. In addition, the definition program designed for this study can be used as a basis for further development of vocabulary intervention studies and for discussion with and among educators working with Spanish-speaking populations.

## Supporting information

S1 Appendix List of intervention words in the order they were taught. *Note*. Gram. Class = grammatical class (A = adjective, N = noun, V = verb); Freq/million = frequency of appearance per million words in written material (Martínez-Martín & García, 2004); Richness = number of different meanings; Productivity = number of derivatives.
(DOCX)

S2 Appendix List of control words in alphabetical order. *Note*. Gram. Class = grammatical class (A = adjective, N = noun, V = verb); Freq/million = frequency of appearance per million words in written material (Martínez-Martín & García, 2004); Richness = number of different meanings; Productivity = number of derivatives.
(DOCX)

S3 Appendix Resumen en español (short summary in Spanish). *Note*. Referencia para el texto completo en inglés: Gomes-Koban, Simpson, Valle, & Defior. Oral vocabulary training program for Spanish third-graders with low socio-economic status: A randomized controlled trial.
(DOCX)

## References

[1] Baumann JF. Vocabulary and reading comprehension: The nexus of meaning In: Israel SE, Duffy GG, editors. Handbook of Research on Reading Comprehension. New York, NY: Routledge; 2009 pp. 323–346.

[2] Biemiller A, Boote C. An effective method for building meaning vocabulary in primary grades. J Educ Psychol, 2006; 98(1): 44–62. doi: 10.1037/0022-0663.98.1.44

[3] Hart B, Risley TR. The early catastrophe: The 30 million word gap by age 3. American Educator 2003: 4–9. Available from: https://www.aft.org/sites/default/files/periodicals/TheEarlyCatastrophe.pdf

[4] Perfetti CA, Landi N, Oakhill J. The acquisition of reading comprehension skill In: Snowling MJ, Hulme C, editors. The science of reading: A handbook. Oxford, UK: Blackwell; 2007 pp. 227–247.

[5] Fernald A, Marchman VA, Weisleder A. SES differences in language processing and vocabulary are evident at 18 months. Dev Sci. 2013; 16(2): 234–248. doi: 10.1111/desc.12019 23432833 10.1111/desc.12019 PMC3582035

[6] White TG, Graves MF, Slater WH. Growth of reading vocabulary in diverse elementary schools: Decoding and word meaning. J Educ Psychol. 1990; 82(2): 281–290.

[7] Organisation for Economic Co-operation and Development. PISA 2015 Results: Excellence and equity in education (Vol. I); 2015. Available from http://www.keepeek.com/Digital-Asset-Management/oecd/education/pisa-2015-results-volume-i_9789264266490-en#page1. doi: 10.1787/9789264266490-en

[8] Justicia FJ. El desarrollo del vocabulario: diccionario de frecuencias [Vocabulary development: Frequency dictionary]. Granada: Servicio de Publicaciones. Universidad de Granada; 1995.

[9] Silva SM, Verhoeven L, van Leeuwe J. Socio-cultural predictors of reading literacy in fourth graders in Lima, Peru. Writ Lang Lit. 2008, 11(1): 15–34.

[10] National Institute of Child Health Development. Report of the National Reading Panel. Teaching Children to Read: An evidence-based assessment of the scientific research literature on reading and its implications for reading instruction: Reports of the subgroups. NIH Publication No. 00.4754. Washington, DC: U.S. Government Printing Office; 2000. Available from: https://www.nichd.nih.gov/publications/pubs/nrp/documents/report.pdf

[11] Snow C. Reading for Understanding: Toward an R&D program in reading comprehension. Washington, DC: The RAND Corporation; 2002 Available from: https://www.rand.org/content/dam/rand/pubs/monograph_reports/2005/MR1465.pdf

[12] Butler S, Urrutia K, Buenger A, Gonzalez N, Hunt M, Eisenhart C. A review of the current research on vocabulary instruction. National Reading Technical Assistance Center, RMC Research Corporation; 2010 Available from https://www2.ed.gov/programs/readingfirst/support/rmcfinal1.pdf

[13] Beck IL, McKeown MG, Kucan L. Bringing words to life: Robust vocabulary instruction. New York: Guilford; 2002.

[14] Wendling BJ, Mather N. Essentials of evidence-based academic interventions. Hoboken, NJ: John Wiley & Sons; 2009.

[15] Ferrándiz-Mingot J. Vocabulario Común y Fundamental. Vida Escolar 1978: 197–198.

[16] Pelatti CY, Piasta SB, Justice LM, O’Connell A. Language- and literacy-learning opportunities in early childhood classrooms: Children’s typical experiences and within-classroom variability. Early Child Res Q. 2014, 29: 445–456. http://dx.doi.org/10.1016/j.ecresq.2014.05.004

[17] Angulo-Domínguez MC, Ocampos JG, Luque-Vilaseca JL, Rodríguez-Romero MP, Sánchez-Cantero R, Satorras-Fioretti R M, Vázquez-Uceda M. Manual de atención al alumnado con necesidades específicas de apoyo educativo derivadas de dificultades específicas de aprendizaje: dislexia. [Manual for students with special educational needs derived from specific learning difficulties: dyslexia] Junta de Andalucía—Consejería de Educación; 2011 Available from http://www.juntadeandalucia.es/educacion/www/portal/com/bin/Contenidos/PSE/orientacionyatenciondiversidad/educacionespecial/guiadislexia/1328017760576_dislexia.pdf

[18] Vasco Gobierno. Guía de buenas prácticas. El profesorado ante la enseñanza de la lectura. [Guide of good practices. Teachers facing the teaching of reading]. Departamento de Educación, Universidades e Investigación; 2006 Available from http://www.hezkuntza.ejgv.euskadi.net/r43-573/es/contenidos/informacion/dia6/es_2027/adjuntos/RecursosParaLaInclusion/ensenanza_lectura_c.pdf

[19] Ministerio de Educación, Cultura y Deporte de España, Centro Nacional de Innovación e Investigación Educativa. La atención al alumnado con dislexia en el sistema educativo en el contexto de las necesidades específicas de apoyo educativo (Colección Eurydice España-RediE). [Special program for students with dyslexia in the regular educational system in the context of specific educational needs]; 2012. Available from http://sid.usal.es/idocs/F8/FDO26768/atencion_alumnado_dislexia.pdf

[20] Seymour PHK, Aro M, Erskine JM. Foundation literacy acquisition in European orthographies. Br J Psychol. 2003, 94(2): 143–174. doi: 10.1348/000712603321661859 12803812 10.1348/000712603321661859

[21] Dauer RM. Stress-timing and syllable-timing reanalyzed. J Phon. 1983, 11: 51–62.

[22] Calet N, Gutiérrez-Palma N, Simpson IC, González-Trujillo MC, Defior S. Suprasegmental phonology development and reading acquisition: A longitudinal study. Sci Stud Read. 2015, 19(1): 51–71. doi: 10.1080/10888438.2014.976342

[23] Carreiras M, Perea M. Naming pseudowords in Spanish: Effects of syllable frequency. Brain Lang. 2004, 90: 393–400. doi: 10.1016/j.bandl.2003.12.003 15172555 10.1016/j.bandl.2003.12.003

[24] Share DL. On the Anglocentricities of current reading research and practice: the perils of overreliance on an «outlier» orthography. Psychol Bull. 2008, 134(4): 584–615. doi: 10.1037/0033-2909.134.4.584 18605821 10.1037/0033-2909.134.4.584

[25] Manolitsis G, Georgiou G, Stephenson K, Parrila R. Beginning to read across languages varying in orthographic consistency: Comparing the effects of non-cognitive and cognitive predictors. Learn Instr. 2009, 19: 466–480. doi: 10.1016/j.learninstruc.2008.07.003

[26] Morales FM. Eficacia de un programa de entrenamiento en el vocabulario en niños. Revista de Investigación en Logopedia. 2013; 3: 1–17.

[27] Pérez RG. Enseñanza de estrategias para la inferencia del significado de las palabras. Infancia y Aprendizaje. 1995, 18(72): 139–152. doi: http://dx.doi.org/10.1174/02103709560561203

[28] Larraín A, Strasser K, Lissi MR. Lectura compartida de cuentos y aprendizaje de vocabulario en edad preescolar: un estudio de eficacia. [Shared storybook reading and vocabulary learning in preschoolers: An effectiveness study]. Estudios de Psicología. 2012, 33(3): 379–383.

[29] Nagy WE, Scott JA. Vocabulary processes In: Kamil ML, Mosenthal P, Pearson PD, Barr R, editors. Handbook of reading research. volume 3. Mahwah, NJ: Erlbaum; 2000 pp. 269–284.

[30] Nagy WE, Anderson RC, Herman PA. Learning word meanings from context during normal reading. Am Educ Res J. 1987; 24(2): 237–270.

[31] Graves MF. The vocabulary book: learning and instruction. New York, NY: Teachers College Press; 2006.

[32] Carlisle J F, Fleming JE, Gudbrandsen B. Incidential word learning in science classes. Contemp Educ Psychol. 2000; 25(2): 184–211. doi: 10.1006/ceps.1998.1001 10753546 10.1006/ceps.1998.1001

[33] Kuhn MR, Stahl SA. Teaching children to learn word meanings from context: A synthesis and some questions. J Lit Res. 1998; 30(1): 119–138.

[34] Jenkins JR, Stein ML, Wysocki K. Learning vocabulary through reading. Am Educ Res J. 1984; 21(4): 767–787.

[35] Beck IL, McKeown MG. Text Talk: Capturing the benefits of read-aloud experiences for young children. Read Teach. 2001; 55(1): 10–20.

[36] Marulis LM, Neuman SB. The effects of vocabulary intervention on young children’s word learning: A meta-analysis. J Lit Res. 2010; 80(3): 300–335. doi: 10.3102/0034654310377087

[37] Chall JS. Two vocabularies for reading: Recognition and meaning In: McKeown MG, Curtis ME, editors. The Nature of Vocabulary Acquisition. Hillsdale, NJ: LEA; 1987 pp. 7–17.

[38] Perfetti CA. Reading ability: Lexical quality to comprehension. Sci Stud Read. 2007; 11(4): 357–383. doi: 10.1080/10888430701530730

[39] Biemiller A. Vocabulary: Needed if more children are to read well. Read Psychol. 2003; 24: 323–335. doi: 10.1080/02702710390227297

[40] Beck IL, McKeown MG, Omanson RC. The effects and uses of vocabulary instructional techniques In: McKeown MG, Curtis ME, editors. The Nature of Vocabulary Acquisition. Hillsdale, NJ: Erlbaum; 1987 pp. 147–163.

[41] Cronbach LJ. An analysis of techniques for diagnostic vocabulary testing. J Educ Res. 1942; 36: 206–217.

[42] Dale E. Vocabulary measurement: Techniques and major findings. Elementary English. 1965; 42: 82–88.

[43] Perfetti CA, Hart L. The lexical bases of comprehension skill In: Gorfien DS, editor. On the consequences of meaning selection: Perspectives on resolving lexical ambiguity. Washington, DC: APA; 2001 pp. 67–86.

[44] Calfee RC, Drum PA. Research on teaching reading In: Wittrock MD, editor. Handbook of research on teaching. 3rd ed. New York: Macmillan; 1986 pp. 804–849.

[45] Beck IL, Perfetti CA, McKeown MG. Effects of long-term vocabulary instruction on lexical access and reading comprehension. J Educ Psychol. 1982; 74(4): 506–521.

[46] Beck IL, McKeown MG. Increasing young low-income children’s oral vocabulary repertoires through rich and focused instruction. Elem Sch J. 2007; 107: 251–273.

[47] Fawcett AJ, Nicholson RI. Vocabulary training for children with dyslexia. J Learn Disabil–Research Briefs. 1991; 24(6): 379–383. 10.1177/002221949102400609 1940598

[48] McKeown MG, Beck IL, Omanson RC, Perfetti CA. The effects of long-term vocabulary instruction on reading comprehension: A replication. J Read Behav. 1983; 15(1): 3–18.

[49] McKeown MG, Beck IL, Omanson RC, Pople MT. Some effects of the nature and frequency of vocabulary instruction on the knowledge and use of words. Read Res Q. 1985; 20(5): 522–534.

[50] Graves MF, Watts-Taffe SM. The place of word consciousness in a research-based vocabulary program In: Samuels SJ, Farstrup AE, editors. What research has to say about reading instruction. 3rd ed. Newark, DE: IRA; 2002 pp. 140–165.

[51] Stahl SA, Nagy WE. Teaching word meanings–Literacy Teaching Series. 2nd ed. New York: Routledge; 2012.

[52] Nagy WE. Metalinguistic awareness and the vocabulary-comprehension connection In: Wagner RK, Muse AE, Tannenbaum KR, editors. Vocabulary Acquisition: Implications for Reading Comprehension. New York, NY: Guilford; 2007 pp. 57–77.

[53] Elleman AM, Lindo EJ, Morphy P, Compton DL. The impact of vocabulary instruction on passage-level comprehension of school-age children: A meta-analysis. J Res Educ Eff. 2009; 2: 1–44. doi: 10.1080/19345740802539200

[54] Stahl SA, Fairbanks MM. The effects of vocabulary instruction: A model-based meta-analysis. Rev Educ Res. 1986; 56(1): 72–110.

[55] Taylor WL. Cloze procedure: A new tool for measuring readability. Journal Q. 1953; 30: 415–433.

[56] Nash H, Snowling M. Teaching new words to children with poor existing vocabulary knowledge: a controlled evaluation of the definition and context methods. Int J Lang Commun Disord. 2006; 41(3): 335–354. doi: 10.1080/13682820600602295 16702097 10.1080/13682820600602295

[57] Nelson JR, Stage SA. Fostering the development of vocabulary knowledge and reading comprehension through contextually-based multiple meaning vocabulary instruction. Educ Treat Children. 2007, 30(1): 1–22.

[58] Jenkins JR, Matlock B, Slocum TA. Two approaches to vocabulary instruction: the teaching of individual word meanings and practice in deriving word meanings from context. Read Res Q. 1989; 24(2): 215–235.

[59] Beck IL, McKeown MG. Conditions of vocabulary acquisition In: Barr R, Kamil ML, Mosenthal PB, Pearson PD, editors. Handbook of Reading Research. volume 2. White Plains, NY: Longman; 1996 pp.789–810.

[60] Oakhill J, Cain K. Introduction to comprehension development In: Cain K, Oakhill J, editors. Children’s Comprehension Problems in Oral and Written Language: A cognitive perspective. New York, NY: Guilford; 2007 pp. 3–40.

[61] Clarke PJ, Snowling MJ, Truelove E, Hulme C. Ameliorating children’s reading comprehension difficulties: A randomized controlled trial. Psychol Sci. 2010; 21(8): 1106–1116. doi: 10.1177/0956797610375449 20585051 10.1177/0956797610375449

[62] Rose J. Independent review of the teaching of early reading: Final report. Nottingham: Department for Education and Skills Publications; 2006 Available from: http://dera.ioe.ac.uk/5551/2/report.pdf

[63] Karten TJ. Inclusive Practices. Thousand Oaks, CA: Corwin; 2011.

[64] Faul F, Erdfelder E, Lang AG, Buchner A. G*Power 3: A flexible statistical power analysis program for the social, behavioral, and biomedical sciences. Behav Res Methods. 2007; 39(2): 175–191. doi: 10.3758/BF03193146 17695343 10.3758/bf03193146

[65] Dunn LM, Dunn LM, Arribas D. Peabody Test de Vocabulário de Imágenes. 3rd ed. Madrid, Spain: TEA; 2006.

[66] Corral S, Arribas D, Santamaría P, Sueiro MJ, Pereña J. Escala de Inteligencia de Wechsler para Niños. 4th ed. Madrid, Spain: TEA; 2005.

[67] Alliende F, Condemarin M, Milicic N. Prueba de Comprensión Lectora de Complejidad Lingüística Progresiva. Madrid, Spain: Ciencias de la Educación Preescolar y Especial; 1991.

[68] Cohen J. A coefficient of agreement for nominal scales. Educ Psychol Meas. 1960; 20: 37–46.

[69] Fleiss JL, Cohen J. The equivalence of weighted kappa and the intraclass correlation coefficient as measures of reliability. Educ Psychol Meas.1973; 33: 613–619.

[70] Cohen J. Statistical Power Analysis for the Behavioral Sciences. 2nd ed. New York, NY: Psychology Press; 1988.

[71] Martínez-Martín JA, García-Pérez E. Diccionario: Frecuencias del catellano escrito en niños de 6 a 12 años. Salamanca: Publications Universidad Pontificia; 2004.

[72] Cepeda NJ, Pashler H, Vul E, Wixted JT, Rohrer D. Distributed practice in verbal recall tasks: A review and quantitative synthesis. Psychol Bull. 2006; 132(3): 354–380. doi: 10.1037/0033-2909.132.3.354 16719566 10.1037/0033-2909.132.3.354

[73] Mason LH. Explicit self-regulated strategy development versus reciprocal questioning: Effects on expository reading comprehension among struggling readers. J Educ Psychol. 2006; 96(2): 283–296. doi: 10.1037/0022-0663.96.2.283

[74] Mason LH. Teaching students who struggle with learning to think before, while, and after reading: Effects of self-regulated strategy development instruction. Read Writ Q. 2013; 29: 124–144. doi: 10.1080/10573569.2013.758561

[75] Albella N, Fernández-Montijano M. Paso a Paso 3—Comprensión Lectora. Madrid: Anaya; 2006.

[76] Craik FIM, Lockhart RS. Levels of processing: A framework for memory research. J Verbal Learning Verbal Behav. 1972; 11: 671–684.

[77] Marton F, Säljo R. Approaches to learning In: Marton F, Hounsell DJ, Entwistle NJ, editors. The experience of learning. Edinburgh: Scottish Academic Press; 1984 pp. 36–55.

[78] Bandura A. Social Learning Theory. New York: General Learning Press; 1977.

[79] Vygotsky LS. Mind in Society: The development of higher psychological processes Cole M, John-Steiner V, Scribner S, Souberman E, editors. Cambridge, MA: Harvard University Press; 1978 (Original manuscripts [ca. 1930–1934]).

[80] Wood DJ, Bruner JS, Ross G. The role of tutoring in problem solving. J Child Psychol Psychiatry. 1976; 17(2): 89–100. 932126 10.1111/j.1469-7610.1976.tb00381.x

[81] Bluestein J. Avoid win-lose power strategies In: Bluestein J, editor. Classroom Management. Thousand Oaks, CA: Corwin; 2011 pp. 133–146.

[82] Pirangelo R, Giuliani G. Understand the behaviors of students with emotional and/or behavioral disorders In: Bluestein J, editor. Classroom Management. Thousand Oaks, CA: Corwin; 2011 pp. 151–171.

[83] Van Breukelen GJP. ANCOVA versus change from baseline had more power in randomized studies and more bias in nonrandomized studies. J Clin Epidemiol. 2006; 59(9): 920–925. doi: 10.1016/j.jclinepi.2006.02.007 16895814 10.1016/j.jclinepi.2006.02.007

[84] Bates DM, Maechler M, Bolker B, Walker S. lme4: Linear mixed-effects models using Eigen and S4. R package version 1.1.11; 2006 Available from: http://lme4.r-forge.r-project.org/

[85] R Development Core Team. R: A language and environment for statistical computing. R Foundation for Statistical Computing, Vienna, Austria ISBN 3-900051-07-0; 2012 Available from: http://www.R-project.org/

[86] Bates D. lmer, p-values and all that; 2006. Available from: https://stat.ethz.ch/pipermail/r-help/2006-May/094765.html

[87] Baayen RH, Davidson DJ, Bates DM. Mixed-effects modelling with crossed random effects for subjects and items. J Mem Lang. 2008; 59(4): 390–412. doi: 10.1016/j.jml.2007.12.005

[88] Stanovich KE. Matthew effects in reading: Some consequences of individual differences in the acquisition of literacy. Read Res Q. 1986; 21(4): 360–407.

[89] Nagy WE, Herman PA. Breadth and depth of vocabulary knowledge: Implications for acquisition and instruction In: McKeown MG, Curtis ME, editors. The Nature of Vocabulary Acquisition. Hillsdale, NJ: LEA; 1987 pp. 19–35.

[90] Elley WB. Vocabulary acquisition from listening to stories read aloud. Read Res Q. 1989; 24: 174–187.

[91] Gombert JE. Metalinguistic development. Chicago: University of Chicago Press; 1992.

[92] Sternberg R. Beyond IQ: A triarchic theory of human intelligence New York: Cambridge University Press; 1985.

[93] Anderson JR. The architecture of cognition. Cambridge, MA: Harvard University Press; 1983.

[94] Stahl SA. Vocabulary and readability: How knowing word meanings affects comprehension. Top Lang Disord. 2003; 23(3): 241–247.

[95] Walley AC. The role of vocabulary development in children’s spoken word recognition and segmentation ability. Dev Rev. 1993; 13: 286–350.

