# Supplementary material for: Oral vocabulary training program for Spanish third-graders with low socio-economic status: A randomized controlled trial
Source: PLoS One. 2017 Nov 29;12(11):e0188157. doi: 10.1371/journal.pone.0188157 (PMC5706695; doi:10.1371/journal.pone.0188157)
Supplement: S1 Appendix — Note. Gram. Class = grammatical class (A = adjective, N = noun, V = verb); Freq/million = frequency of appearance per million words in written material (Martínez-Martín & García, 2004); Richness = number of different meanings; Productivity = number of derivatives. (DOCX) [file pone.0188157.s001.docx]

**S1 File. List of intervention words in the order they were taught.**

| **Session** | **Words** | **Gram. Class** | **Length** | **Freq/**  **million** | **Richness** | **Productivity** |
| --- | --- | --- | --- | --- | --- | --- |
| 1 | ingenio | N | 7 | 17.68 | 6 | 3 |
|  | atreverse | V | 9 | 8.21 | 1 | 3 |
|  | insoportable | A | 12 | 19.82 | 2 | 2 |
| 2 | aficionado | N | 10 | 15.54 | 3 | 3 |
|  | detectar | V | 8 | 18.93 | 1 | 4 |
|  | fundamental | A | 11 | 48.21 | 2 | 3 |
| 3 | precaución | N | 10 | 9.64 | 2 | 3 |
|  | intervenir | V | 10 | 18.57 | 4 | 4 |
|  | cómodo | A | 6 | 25.89 | 3 | 5 |
| 4 | dignidad | N | 8 | 35.36 | 4 | 4 |
|  | insistir | V | 8 | 12.50 | 3 | 3 |
|  | aplicado | A | 8 | 16.07 | 2 | 6 |
| 5 | amenaza | N | 7 | 47.86 | 2 | 4 |
|  | ocultar | V | 7 | 17.14 | 4 | 4 |
|  | interminable | A | 12 | 15.18 | 1 | 4 |
| 6 | refugio | N | 7 | 21.25 | 2 | 3 |
|  | proponer | V | 8 | 9.11 | 3 | 3 |
|  | adecuado | A | 8 | 60.17 | 1 | 4 |
| 7 | protagonista | N | 12 | 34.11 | 2 | 3 |
|  | empeñarse | V | 9 | 0.54 | 6 | 5 |
|  | denso | A | 5 | 16.08 | 3 | 4 |
| 8 | aparato | N | 7 | 53.93 | 7 | 3 |
|  | proporcionar | V | 12 | 10.71 | 3 | 5 |
|  | trágico | A | 7 | 21.61 | 3 | 2 |
| 9 | rastro | N | 6 | 18.39 | 3 | 2 |
|  | recurso | N | 7 | 27.68 | 4 | 2 |
|  | disponer | V | 8 | 17.32 | 7 | 7 |
|  | siniestro | A | 9 | 19.47 | 5 | 3 |
| 10 | satisfacción | N | 12 | 36.07 | 6 | 6 |
|  | superar | V | 7 | 26.96 | 5 | 4 |
|  | revelar | V | 7 | 7.32 | 3 | 4 |
|  | orgulloso | A | 9 | 20.53 | 2 | 3 |
| 11 | responsabilidad | N | 15 | 63.04 | 2 | 4 |
|  | suceder | V | 7 | 15.71 | 3 | 5 |
|  | repleto | A | 7 | 13.57 | 1 | 1 |
|  | idéntico | A | 8 | 25.36 | 2 | 2 |
| 12 | asombro | N | 7 | 26.07 | 2 | 3 |
|  | casualidad | N | 10 | 23.93 | 2 | 2 |
|  | distinguir | V | 10 | 26.07 | 3 | 6 |
|  | espléndido | A | 10 | 19.82 | 2 | 4 |
| 13 | vértigo | N | 7 | 13.04 | 3 | 2 |
|  | rescatar | V | 8 | 8.21 | 3 | 2 |
|  | conquistar | V | 10 | 8.04 | 4 | 4 |
|  | semejante | A | 9 | 67.14 | 4 | 6 |
| 14 | vigilancia | N | 10 | 22.86 | 2 | 5 |
|  | prever | V | 6 | 10.89 | 2 | 6 |
|  | repentino | A | 9 | 18.93 | 1 | 3 |
|  | apropiado | A | 9 | 15.17 | 1 | 3 |
| 15 | entusiasmo | N | 10 | 43.04 | 3 | 4 |
|  | probabilidad | N | 12 | 15.71 | 2 | 3 |
|  | lograr | V | 6 | 42.50 | 1 | 4 |
|  | procedente | A | 10 | 14.82 | 2 | 4 |
| 16 | estabilidad | N | 11 | 22.50 | 3 | 4 |
|  | contemplar | V | 10 | 34.29 | 4 | 3 |
|  | comprobar | V | 9 | 60.24 | 1 | 3 |
|  | auténtico | A | 9 | 66.43 | 3 | 4 |
| 17 | reconocimiento | N | 14 | 58.21 | 2 | 4 |
|  | detener | V | 7 | 19.29 | 3 | 4 |
|  | severo | A | 6 | 19.46 | 3 | 2 |
|  | pendiente | A | 9 | 31.61 | 7 | 2 |

*Note.* Gram. Class = grammatical class (A = adjective, N = noun, V = verb); Freq/million = frequency of appearance per million words in written material (Martínez-Martín & García, 2004); Richness = number of different meanings; Productivity = number of derivatives.
