# Supplementary material for: Oral vocabulary training program for Spanish third-graders with low socio-economic status: A randomized controlled trial
Source: PLoS One. 2017 Nov 29;12(11):e0188157. doi: 10.1371/journal.pone.0188157 (PMC5706695; doi:10.1371/journal.pone.0188157)
Supplement: S2 Appendix — Note. Gram. Class = grammatical class (A = adjective, N = noun, V = verb); Freq/million = frequency of appearance per million words in written material (Martínez-Martín & García, 2004); Richness = number of different meanings; Productivity = number of derivatives. (DOCX) [file pone.0188157.s002.docx]

**S2 File. List of control words in alphabetical order.**

| **Words** | **Gram. Class** | **Length** | **Freq/**  **million** | **Richness** | **Productivity** |
| --- | --- | --- | --- | --- | --- |
| antigüedad | N | 10 | 15.00 | 4 | 5 |
| concepto | N | 8 | 66.79 | 2 | 6 |
| convertirse | V | 11 | 26.79 | 3 | 7 |
| demostrar | V | 9 | 44.29 | 3 | 4 |
| disimular | V | 9 | 11.79 | 3 | 2 |
| evidente | A | 8 | 69.82 | 1 | 4 |
| experto | A | 7 | 21.07 | 2 | 2 |
| exponer | V | 7 | 8.04 | 4 | 6 |
| fenómeno | N | 8 | 56.61 | 5 | 4 |
| magnífico | A | 9 | 23.04 | 3 | 3 |
| maniobra | N | 8 | 13.04 | 5 | 2 |
| prestigio | N | 9 | 33.75 | 1 | 4 |
| remoto | A | 6 | 27.14 | 3 | 2 |
| suponer | V | 7 | 36.25 | 5 | 4 |
| tierno | A | 6 | 15.89 | 3 | 4 |

*Note.* Gram. Class = grammatical class (A = adjective, N = noun, V = verb); Freq/million = frequency of appearance per million words in written material (Martínez-Martín & García, 2004); Richness = number of different meanings; Productivity = number of derivatives.
