# Supplementary material for: Oral vocabulary training program for Spanish third-graders with low socio-economic status: A randomized controlled trial
Source: PLoS One. 2017 Nov 29;12(11):e0188157. doi: 10.1371/journal.pone.0188157 (PMC5706695; doi:10.1371/journal.pone.0188157)
Supplement: S3 Appendix — Note. Referencia para el texto completo en inglés: Gomes-Koban, Simpson, Valle, & Defior. Oral vocabulary training program for Spanish third-graders with low socio-economic status: A randomized controlled trial. (DOCX) [file pone.0188157.s003.docx]

**S3 Appendix. Resumen en español (short summary in Spanish).**

El conocimiento del vocabulario es un aspecto crucial en el aprendizaje de la lectura (NICHD, 2000; Baumann, 2009). Aunque los niños puedan aprender palabras de forma independiente e implícita, estudios comparativos señalan que hay una ventaja de la enseñanza explícita en comparación a la simple exposición a las palabras sobre todo para aquellos con dificultades de comprensión, baja motivación para la lectura, y los que crecen en un ambiente de lenguaje pobre (Chall, 1987; Marulis y Neuman, 2010; Perfetti, 2007). Los déficits de vocabulario son difíciles de superar y, por lo general, permanecerán a lo largo de toda su carrera académica (Biemiller y Boote, 2006; Fernald, Marchman y Weisleder, 2013; Justicia, 1995; White, Graves y Slater, 1990).

Según Graves (2006), para lograr mejoras en la comprensión lectora, la enseñanza del vocabulario debe contener cuatro elementos básicos: (1) proporcionar experiencias de lenguaje ricas y variadas, (2) enseñar palabras de manera explícita, (3) enseñar estrategias de aprendizaje de palabras, y (4) fomentar la conciencia sobre las palabras. Entre los programas de entrenamiento del vocabulario, existe evidencia sobre métodos con efectos positivos en los niños de habla inglesa, como son el de definición y el de contexto (Beck y McKeown, 1991; Nash y Snowling, 2006), el mapeo semántico (Johnson, Pittelman y Heimlich, 1986) y los sinónimos y antónimos (Graves, Juel y Graves, 2004). Un enfoque igualmente interesante incluye el vocabulario oral como parte de un entrenamiento más amplio de la lengua oral (Clarke, Snowling, Truelove y Hulme, 2010; Snowling y Hulme, 2011).

A pesar de las recomendaciones basadas en evidencia sobre la enseñanza del vocabulario, todavía se observa escasa conexión entre los resultados de la investigación y la práctica en algunos centros escolares en España. Una de las razones de la disparidad entre evidencia y práctica, es el hecho de que, hasta donde llega nuestro conocimiento, no hay programas basados en evidencia y apenas estudios de intervención con niños de habla hispana. Hemos encontrado solamente tres (Larraín, Strasser y Lissi, 2012; Morales, 2013; Pérez, 1995), de los cuales solamente uno ha utilizado una metodología pertinente, como la distribución aleatoria de los niños a los grupos.

Por tanto, este trabajo pretende comprobar la eficacia de dos entrenamientos (definición y contexto), ambos basados en lenguaje oral, para el fomento del vocabulario, en comparación con un grupo control de lectura en voz alta, en una muestra de niños de tercero de EP, de colegios situados en una zona de nivel socioeconómico bajo, y con una cuidadosa metodología. Además, se examinó el potencial de los entrenamientos para fomentar la transferencia del aprendizaje a palabras control no enseñadas y su relación con la comprensión lectora.

Los principales resultados confirman la superioridad de la enseñanza enriquecida del vocabulario, con cualquiera de los dos métodos. Es importante señalar que los niños del grupo control fueron incidentalmente expuestos a las palabras de entrenamiento. Sin embargo, un método como otro fueron más efectivos en comparación con la simple exposición a las palabras del grupo control. La mejor puntuación también en las palabras no enseñadas, mostró que los dos métodos promovían efectos de transferencia de aprendizaje. Una posible explicación sería que, a través de la activación de los conocimientos de las palabras enseñadas, otras palabras y conocimientos semánticos también se activarían (Anderson, 1983) y también la interconexión de los conocimientos sobre las palabras (Aitchison, 2003; Barsalou, 1992; Rumelhart, 1980). Además, en las actividades los niños tuvieron la oportunidad de discutir sobre las palabras, lo que podría haber llevado a un aumento en sus conocimientos semánticos más allá de las palabras enseñadas y, con eso, haber incrementado sus habilidades de conciencia sobre las palabras (Stahl y Nagy, 2012).

Sin embargo, cinco meses después, solamente los niños del grupo definición mostraron mayor conocimiento de las palabras no enseñadas en comparación con los niños del grupo control. Eso sugiere que solo el método definición proporcionó una mejora persistente. Aunque estos resultados no estaban de acuerdo con las hipótesis, la conclusión es que los niños de este grupo no sólo obtuvieron conocimiento semántico, sino que también lograron la capacidad de expresar mejor su conocimiento de las palabras bajo la forma de una definición claramente estructurada, siguiendo un modelo explícito. En contraste, aunque los niños del grupo contexto estuvieron expuestos a más palabras e historias en comparación con los del grupo definición, la forma en que este conocimiento se agregó a las estructuras de conocimiento ya existentes fue menos sistemática. En consecuencia, dependían más de sus propias estrategias de aprendizaje para organizar el conocimiento que se iba presentando.

Debido a que no se encontraron diferencias significativas en las medidas estandarizadas de vocabulario y comprensión lectora, pensamos que los métodos de entrenamiento no lograron un efecto robusto en la conciencia sobre las palabras. En general, los efectos de las intervenciones en vocabulario sobre la comprensión lectora son pequeños y dificiles de detectar (véase meta-análisis de Elleman, Lindo, Morphy y Compton, 2009). En este trabajo, los resultados sugieren que tal vez podría existir un efecto pequeño, pero no tuvo suficiente poder estadístico para revelarlo.

A pesar de algunas limitaciones, este estudio constituye una aportación importante y contribuye a suplir la escasez de estudios en el área de la enseñanza del vocabulario en español. Hasta donde sabemos, es el primer programa de entrenamiento de vocabulario basado en evidencia realizado con niños de habla hispana de educación primaria, utilizando un diseño controlado aleatorizado. Para estudios futuros, pretendemos investigar más detalladamente los efectos de transferencia del método definición en relación con el fomento de la formación de conceptos, así como una estrategia de autoenseñanza para aprender nuevas palabras o para expresar el conocimiento de las palabras, especialmente para niños con dificultades de comprensión del lenguaje y con nivel socioeconómico bajo. La característica estructurada del método podría ayudar a los niños cuando aprenden palabras nuevas de forma independiente, ya que son entrenados para prestar atención a la información específica y para desarrollar mecanismos de almacenamiento de las palabras apoyados por una preestructura. Desde la perspectiva de la práctica, también es un método más transparente y fácil de aplicar por maestros poco experimentados, que podrían apoyarse en las instrucciones dadas en un manual.

Finalmente, se recomienda la utilización del programa de definición como base para el desarrollo de nuevos estudios de intervención en vocabulario y para debatir con los educadores que trabajan con poblaciones de habla hispana.

*Note*. Referencia para el texto completo en inglés: Gomes-Koban, Simpson, Valle, & Defior. Oral vocabulary training program for Spanish third-graders with low socio-economic status: A randomized controlled trial. *PLoSONE*.
